# Restoration, Not Bypass: Otoferlin Gene Therapy and a New Era in Hearing Loss Treatment

**DOI:** 10.1002/ohn.70347

**Published:** 2026-07-18

**Authors:** Jazlyn A. Selvasingh, Justin R. Shinn

**Affiliations:** ^1^ Virginia Tech Carilion School of Medicine Roanoke Virginia USA; ^2^ Carilion Clinic Department of Surgery—Section of Otolaryngology Roanoke Virginia USA

**Keywords:** auditory synaptopathy, cochlear implant, gene therapy, OTOF, otoferlin, sensorineural hearing loss

## Abstract

The FDA approval of Otarmeni, an AAV‐based gene therapy for biallelic OTOF‐associated sensorineural hearing loss, represents a conceptual shift in otology: from bypassing defective auditory physiology to restoring it. Unlike cochlear implantation, which circumvents damaged sensory structures, OTOF gene replacement targets a synaptic deficit in a structurally intact cochlea. Early clinical trial data are encouraging, with 75% of evaluable participants—children with molecularly confirmed biallelic OTOF variants enrolled in early‐phase trials—achieving the primary hearing endpoint at 24 weeks. However, critical questions remain regarding long‐term durability, the feasibility of redosing, and the surgical and logistical infrastructure required for intracochlear delivery. This commentary argues that otoferlin gene therapy initiates a new treatment hierarchy rather than rendering cochlear implantation obsolete: restore when biology permits, bypass when it does not. Realizing this potential will require advances in newborn genetic screening, workforce training, and equitable access to gene delivery.

The FDA approval of Otarmeni (lunsotogene parvec‐cwha), an AAV‐based gene therapy for biallelic OTOF‐associated severe‐to‐profound sensorineural hearing loss, marks a meaningful conceptual shift in otology: from bypassing defective auditory physiology to restoring it.^1^ The FDA indication is specific: patients must have molecularly confirmed biallelic OTOF variants, preserved outer hair cell function, and no prior cochlear implant in the treated ear (Figure 1).

**Figure 1:**
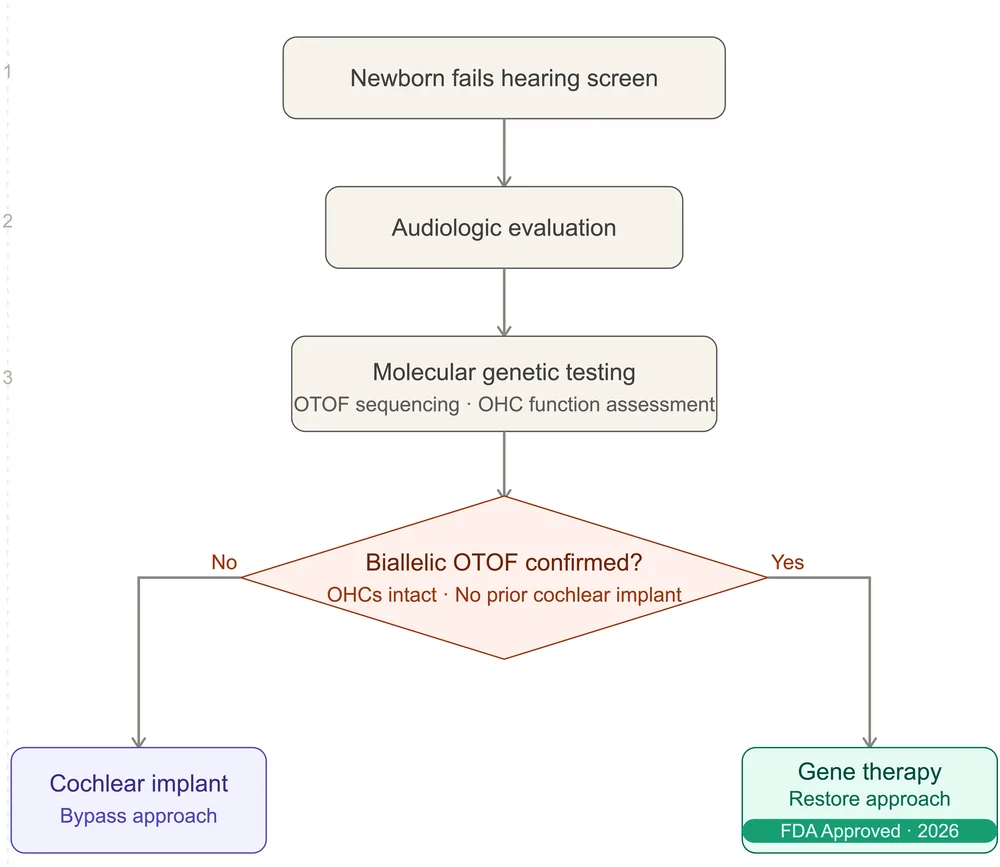
Clinical decision algorithm for gene therapy candidacy in sensorineural hearing loss. Patients who fail newborn hearing screening undergo audiologic evaluation and molecular genetic testing. Those with confirmed biallelic OTOF variants, intact outer hair cell function, and no prior cochlear implant in the treated ear may be candidates for gene therapy (Otarmeni, FDA approved 2026). All others are referred for cochlear implantation. For this molecularly defined subset, restoration now precedes rather than follows device‐based rehabilitation. OHC, outer hair cell.

This specificity reflects the biology. OTOF‐related hearing loss is an auditory synaptopathy in which cochlear architecture may remain intact, but synaptic transmission between inner hair cells and the auditory nerve is disrupted.^2^ The ear is not absent; the signal is (Figure 2). Gene replacement can therefore restore a missing molecular link rather than regenerate a destroyed sensory organ.

**Figure 2:**
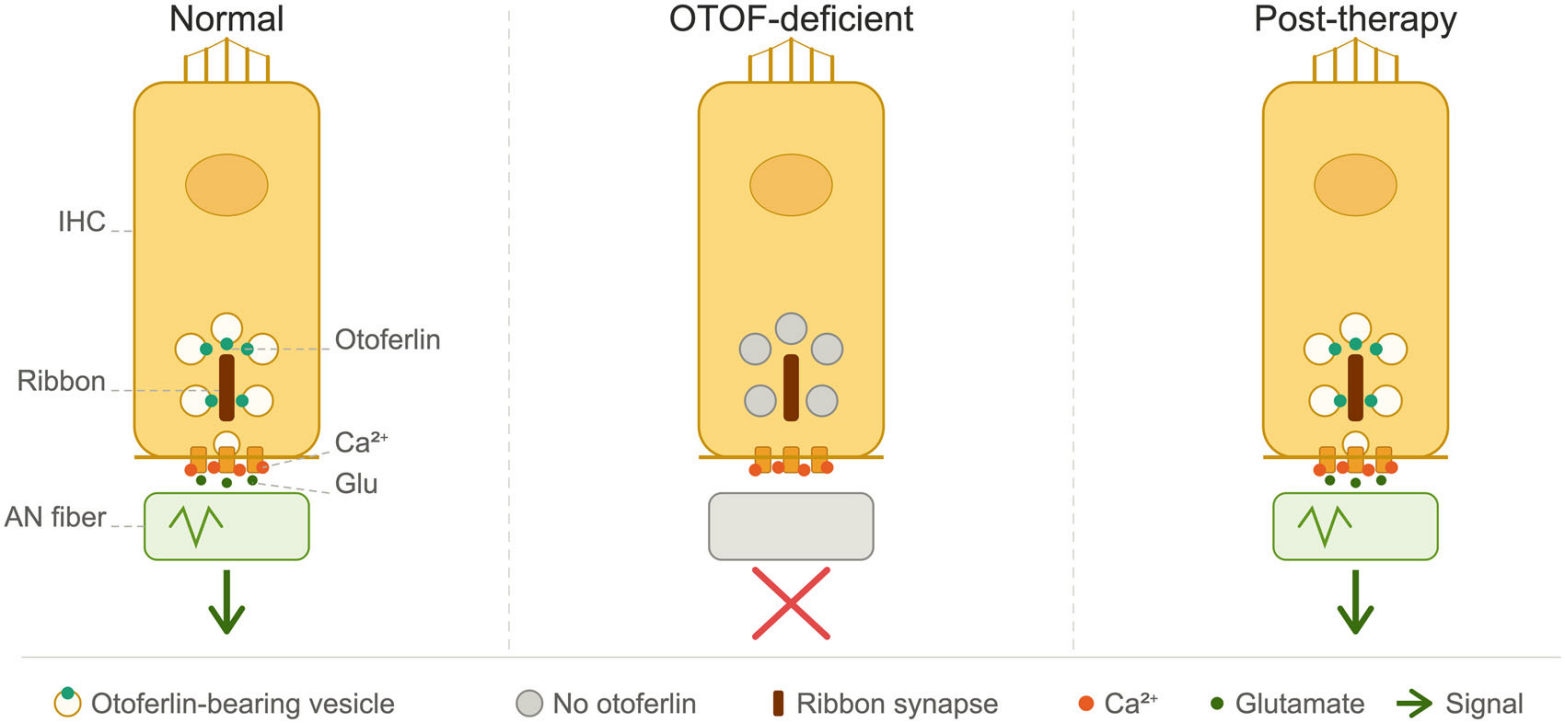
Synaptic transmission at the inner hair cell (IHC) basal pole in three conditions. In the normal state (left), otoferlin on synaptic vesicles senses Ca^2+^ influx through CaV1.3 channels, triggering vesicle fusion at the ribbon synapse and glutamate release onto the auditory nerve fiber. In OTOF‐deficiency (center), Ca^2+^ enters normally, but otoferlin is absent; vesicles cannot fuse, and no signal is transmitted. Following gene therapy (right), otoferlin expression is restored, vesicle fusion resumes, and auditory nerve signaling is re‐established. AN, auditory nerve; Glu, glutamate.

## Clinical Evidence

Early clinical results are encouraging. In the CHORD trial, 75% of evaluable participants achieved the primary hearing endpoint at 24 weeks, and some children reached normal‐hearing thresholds, including detection of whisper‐level sound.^3^ These gains were not observed in the natural history of untreated OTOF‐related deafness. The CHORD cohort consisted of pediatric participants with genetically confirmed biallelic OTOF variants. Generalizability beyond this molecularly defined subset, therefore, remains to be demonstrated, but the magnitude of effect within it is striking.

## Durability and Redosing

Critical questions about durability remain open. Current follow‐up data are limited, and it is not yet known how long functional benefit will be sustained or whether diminishing transgene expression will ultimately necessitate redosing.^4^ AAV‐mediated gene therapy has generally shown durable effects in other tissues, but the cochlear environment, immunological considerations, and the longevity of hair cell transduction require careful longitudinal study. Whether redosing is biologically feasible, and whether prior AAV exposure precludes it, may prove important to pharmaceutical strategy and long‐term treatment planning for patients who receive this therapy early in life.^5^

## Implications for Cochlear Implantation

The implication of this approval is not that cochlear implantation is obsolete. Rather, this approval represents an inversion of the current treatment algorithm for a narrow but meaningful subset of patients—those with biallelic OTOF variants and preserved cochlear architecture—for whom restoration now precedes, rather than follows, device‐based rehabilitation. It remains indispensable when sensory cells, synapses, or neural structures cannot be biologically rescued. Yet for selected genetic forms of deafness, implantation may increasingly become a second‐line option rather than the default endpoint of care.

## Surgical Delivery and Workforce Preparation

This approval raises practical questions about surgical delivery and workforce preparation. Otarmeni is administered via intracochlear injection, a procedure that shares anatomic territory with cochlear implantation but represents a distinct surgical task.^6^ Unlike cochlear implantation, intracochlear gene delivery requires controlled vector distribution within an intact cochlea, where even minor mechanical trauma may compromise both baseline function and therapeutic efficacy. Reliable access to the scala tympani, precise injection rate and volume control, and predictable apical‐to‐basal dose distribution are central to outcomes, and small deviations in technique may translate into measurable differences in transduction efficiency. Whether this will be performed by cochlear implant surgeons, neurotologists, or general otolaryngologists with additional training is not yet defined. The learning curve, credentialing pathways, and required equipment will need to be established at the program level, and the distribution of this expertise could meaningfully shape access. If delivery is concentrated in high‐volume academic centers, patients in underserved regions may face geographic barriers not unlike those that already limit timely cochlear implantation.^7^

## Diagnostic Infrastructure

This approval also exposes a diagnostic bottleneck. Universal newborn hearing screening identifies hearing loss, but not molecular etiology. A therapy whose benefit depends on preserved cochlear biology demands rapid genetic confirmation, early referral pathways, and centers capable of both interpretation and intracochlear delivery. Without this infrastructure, restoration will remain technically possible but clinically delayed (Figure 3).

**Figure 3:**
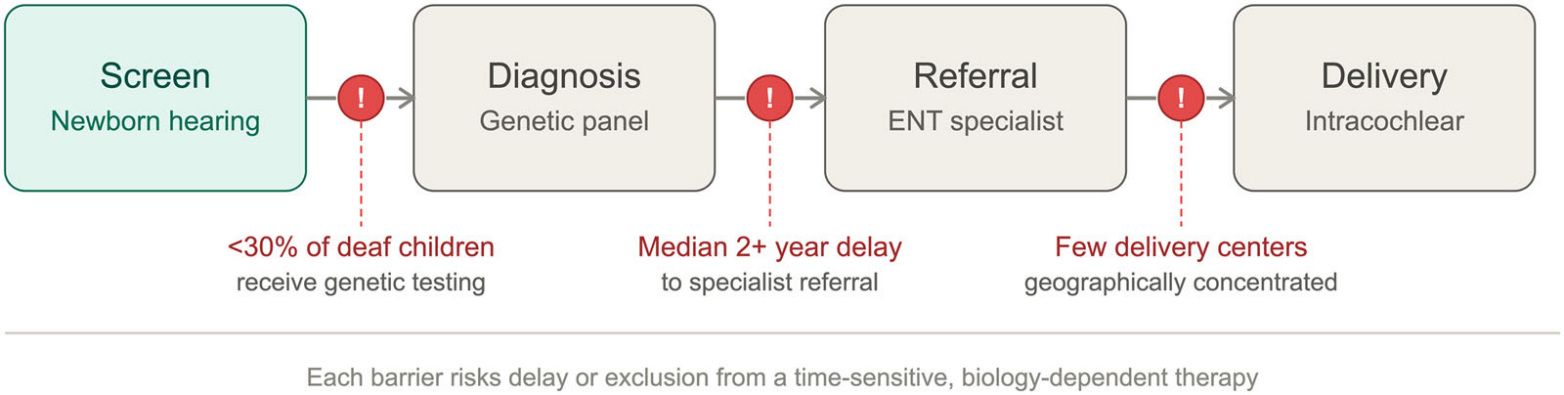
Care pathway bottlenecks between newborn hearing screen and gene therapy delivery. Universal newborn hearing screening identifies hearing loss reliably, but fewer than 30% of deaf children subsequently receive genetic testing. Those who do face median delays exceeding two years before specialist referral, and intracochlear gene delivery remains concentrated in a small number of academic centers. Each barrier risks delaying or excluding patients from a time‐sensitive, biology‐dependent therapy.

## Looking Ahead

The next frontier will be harder. OTOF is monogenic and biologically favorable. Targets such as GJB2, STRC, and acquired cochlear synaptopathies will test whether this success can generalize beyond a rare, structurally preserved phenotype. Preclinical work has already demonstrated auditory rescue in GJB2 and STRC models, while neurotrophin‐based strategies continue to explore cochlear synapse repair^8^ (Figure 4). The pricing model also warrants attention: Regeneron has announced that Otarmeni will initially be provided free in the United States, raising a central question about whether access will ultimately be constrained by the same systems that already limit timely genetic testing, specialty referral, and cochlear implantation.^9^ Whether the initial free‐access model can transition into a sustainable paradigm remains uncertain. Gene therapies carry development and manufacturing costs that have historically translated into list prices well above conventional surgical interventions, and no durable framework yet exists for absorbing these costs across payers, health systems, and rare‐disease populations. Without an explicit transitional access strategy, even therapies launched with broad initial coverage risk narrowing access once introductory programs end.

**Figure 4:**
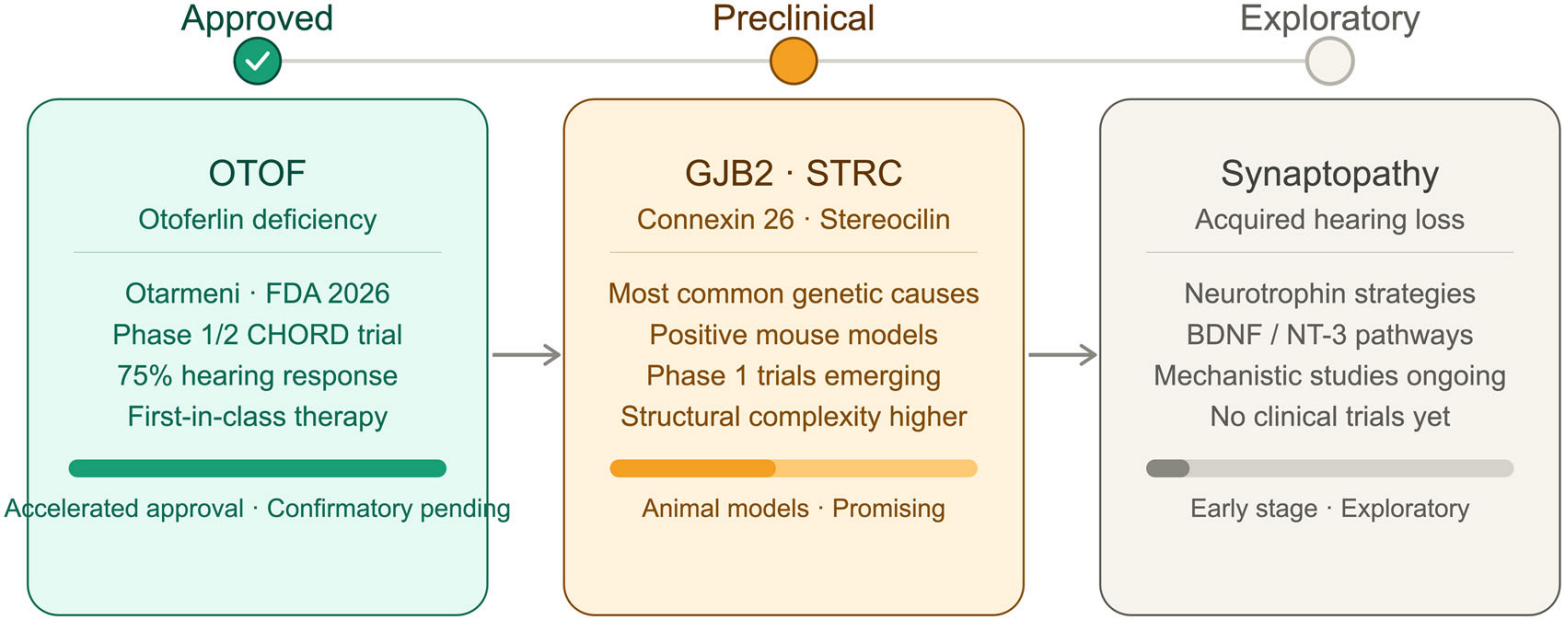
Gene therapy readiness landscape for hereditary hearing loss. OTOF‐related deafness has achieved accelerated FDA approval based on the Phase 1/2 CHORD registrational trial. GJB2 and STRC, the most common genetic causes of hearing loss, have demonstrated proof‐of‐concept in animal models with phase 1 trials emerging. Acquired cochlear synaptopathy remains in early mechanistic exploration. Evidence bars reflect the current strength of clinical and preclinical data for each target.

## Conclusion

Otoferlin gene therapy is an early signal of a broader transition, one that challenges otology to evolve from device‐based rehabilitation toward molecularly guided restoration, and to build the diagnostic, surgical, and access infrastructure that such a transition demands.^10^

## Author contributions

**Jazlyn A. Selvasingh**, conceptualization, literature review, writing—original draft, figure design and preparation; **Justin R. Shinn**, conceptualization, critical revision of the manuscript, writing—review and editing.

## Disclosures

### Competing interests

None declared.

### Funding source

None.
